# Accuracy of deep learning-based integrated tooth models by merging intraoral scans and CBCT scans for 3D evaluation of root position during orthodontic treatment

**DOI:** 10.1186/s40510-022-00410-x

**Published:** 2022-05-09

**Authors:** Suk-Cheol Lee, Hyeon-Shik Hwang, Kyungmin Clara Lee

**Affiliations:** 1 Gwangju, Korea; 2 Department of Orthodontics, School of Dentistry, Chonnam National University, 33 Yongbong-ro, Buk-gu, Gwangju, 61186 Korea

**Keywords:** Deep learning, Tooth models, Intraoral scans, CBCT, Root position, Artificial intelligence

## Abstract

**Objective:**

This study aimed to evaluate the accuracy of deep learning-based integrated tooth models (ITMs) by merging intraoral scans and cone-beam computed tomography (CBCT) scans for three-dimensional (3D) evaluation of root position during orthodontic treatment and to compare the fabrication process of integrated tooth models (ITMs) with manual method.

**Material and methods:**

Intraoral scans and corresponding CBCT scans before and after treatment were obtained from 15 patients who completed orthodontic treatment with premolar extraction. A total of 600 ITMs were generated using deep learning technology and manual methods by merging the intraoral scans and CBCT scans at pretreatment. Posttreatment intraoral scans were integrated into the tooth model, and the resulting estimated root positions were compared with the actual root position at posttreatment CBCT. Discrepancies between the estimated and actual root position including average surface differences, arch widths, inter-root distances, and root axis angles were obtained in both the deep learning and manual method, and these measurements were compared between the two methods.

**Results:**

The average surface differences of estimated and actual ITMs in the manual method were 0.02 mm and 0.03 mm for the maxillary and mandibular arches, respectively. In the deep learning method, the discrepancies were 0.07 mm and 0.08 mm for the maxillary and mandibular arches, respectively. For the measurements of arch widths, inter-root distances, and root axis angles, there were no significant differences between estimated and actual models both in the manual and in the deep learning methods, except for some measurements. Comparing the two methods, only three measurements showed significant differences. The procedure times taken to obtain the measurements were longer in the manual method than in the deep learning method.

**Conclusion:**

Both deep learning and manual methods showed similar accuracy in the integration of intraoral scans and CBCT images. Considering time and efficiency, the deep learning automatic method for ITMs is highly recommended for clinical practice.

## Introduction

Three-dimensional (3D) digital datasets have become the standard. As previous studies have reported the integration of different imaging modalities [1–3], the procedure of treatment planning and evaluation in orthodontics and maxillofacial surgery have shifted to a digital 3D method. However, a cone-beam computed tomography (CBCT) scan has a limitation of occlusal visualization. Most researchers and clinicians agree that CBCT scans do not provide enough detailed information about dentition and interocclusal relationships for treatment planning, owing to the limited scanning resolution and streak artifacts caused by radiopaque dental restorations or orthodontic braces [4–9]. Moreover, taking CBCT scans during or after treatment puts patients and clinicians at risk of radiation exposure. Repeated CBCT scans would expose the patient to higher levels of radiation; this is not recommended clinically, especially in children [10–12].

Lee et al. [13, 14] reported a method for monitoring of root movement with a combination of CBCT at pretreatment and laser-scanned model at posttreatment. The method used in their study is time-consuming and requires much effort. Particularly, the process of individual tooth root isolation from alveolar bone in CBCT is technique sensitive and user dependent. Considering the resolution and noise of images and touching adjacent tooth, touching tooth and bones, tooth segmentation using manual threshold adjustment [15] and region growing [13] might be challenging when handling complex image conditions as well as root-branching problems. Considering that the tooth root isolation is an important step in tooth model fabrication, accurate segmentation is essential. Moreover, a method that isolates the tooth including the root from CBCT images without removing the alveolar bone is preferable. This study aimed to evaluate the accuracy of 3D virtual tooth models and compare deep learning and manual methods in terms of the fabrication process.

## Material and methods

**Fig. 1:**
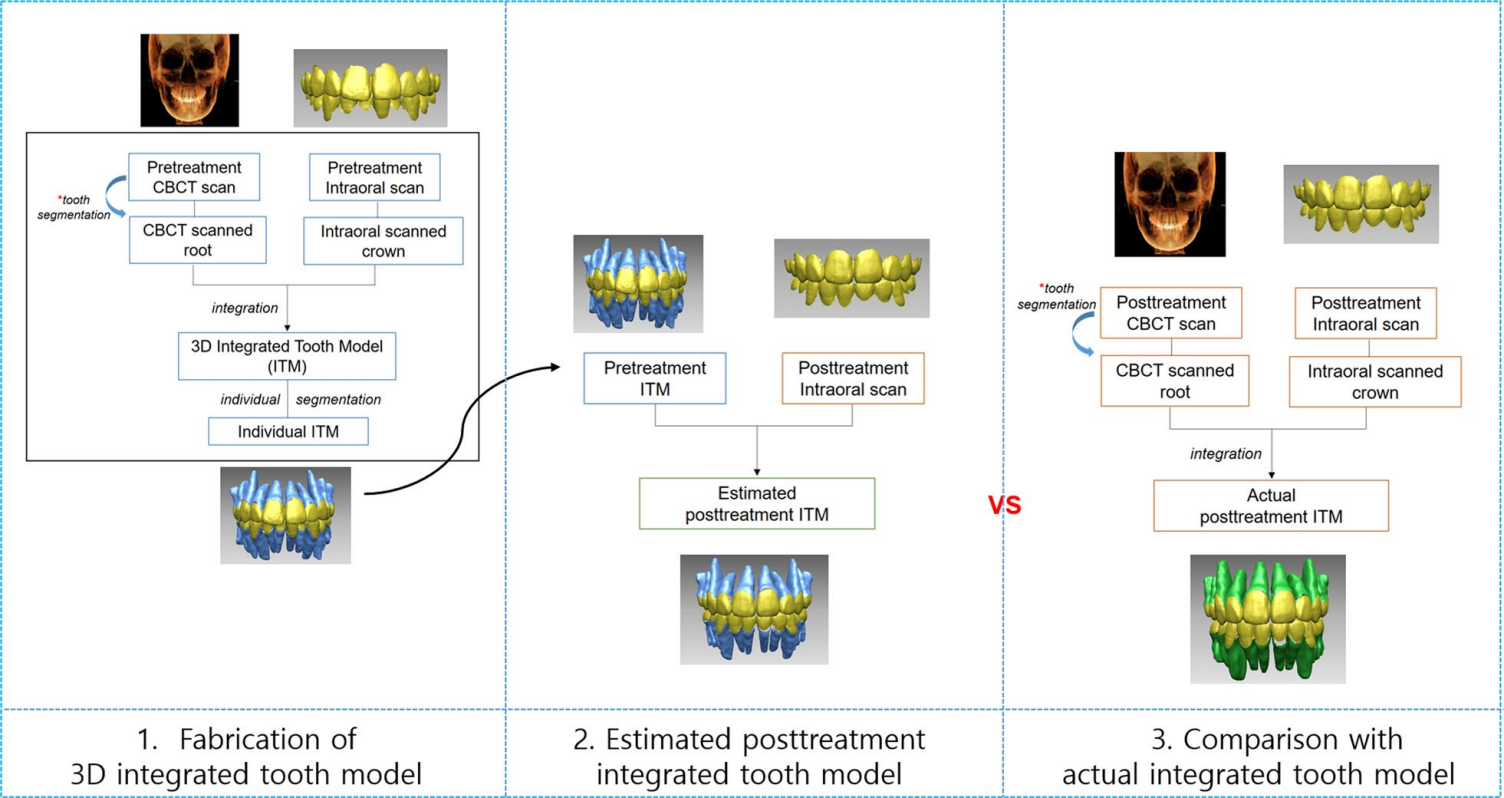
Application scenarios for this study. 3D integrated tooth model was fabricated by merging pretreatment CBCT scan and pretreatment intraoral scans. The fabricated pretreatment integrated tooth model was registered onto the posttreatment intraoral scans and resulting estimated posttreatment tooth model was compared to actual posttreatment tooth models

This study was approved by the institutional review board of Chonnam National University, Gwangju, Korea (CNUDH-EXP-2019–018), in compliance with the principles of the Declaration of Helsinki. Patient records for this study were obtained from the patient database at the Department of Orthodontics at Chonnam National University Dental Hospital. The study protocol is shown in Fig. 1. The inclusion criteria were as follows: (1) patients who have completed orthodontic treatment with premolar extraction and had intraoral scans and (2) patients with available intraoral CBCT scans at the pre- and posttreatment stages. The exclusion criteria were as follows: (1) patients who have undergone restorative treatments during orthodontic treatment; (2) patients who had restorative or prosthodontic treatment covering more than two surfaces during treatment; (3) patients who were treated with interproximal reduction during the treatment; and (4) patients who have dilacerated roots or severe root resorptions.

The sample size calculation was performed according to the results of a study by Lee et al. [14]. In their study, mean difference of buccolingual inclination measurements was 1.30 ± 0.92. The effect size was calculated at 1.41. A statistical power and type I error of 80% and 5%, respectively, were assumed using G*power (version 3.1.9.2, Heinrich Heine University, Dusseldorf, Germany). The calculation indicated that five individuals were required in the study. In this study, 15 patients were included. A total of 600 teeth (300 teeth with central and lateral incisors, canines, second premolar, and first molars in the pretreatment stage; 300 teeth with central and lateral incisors, canines, second premolar, and first molars in the posttreatment stage) were included.

**Fig. 2:**
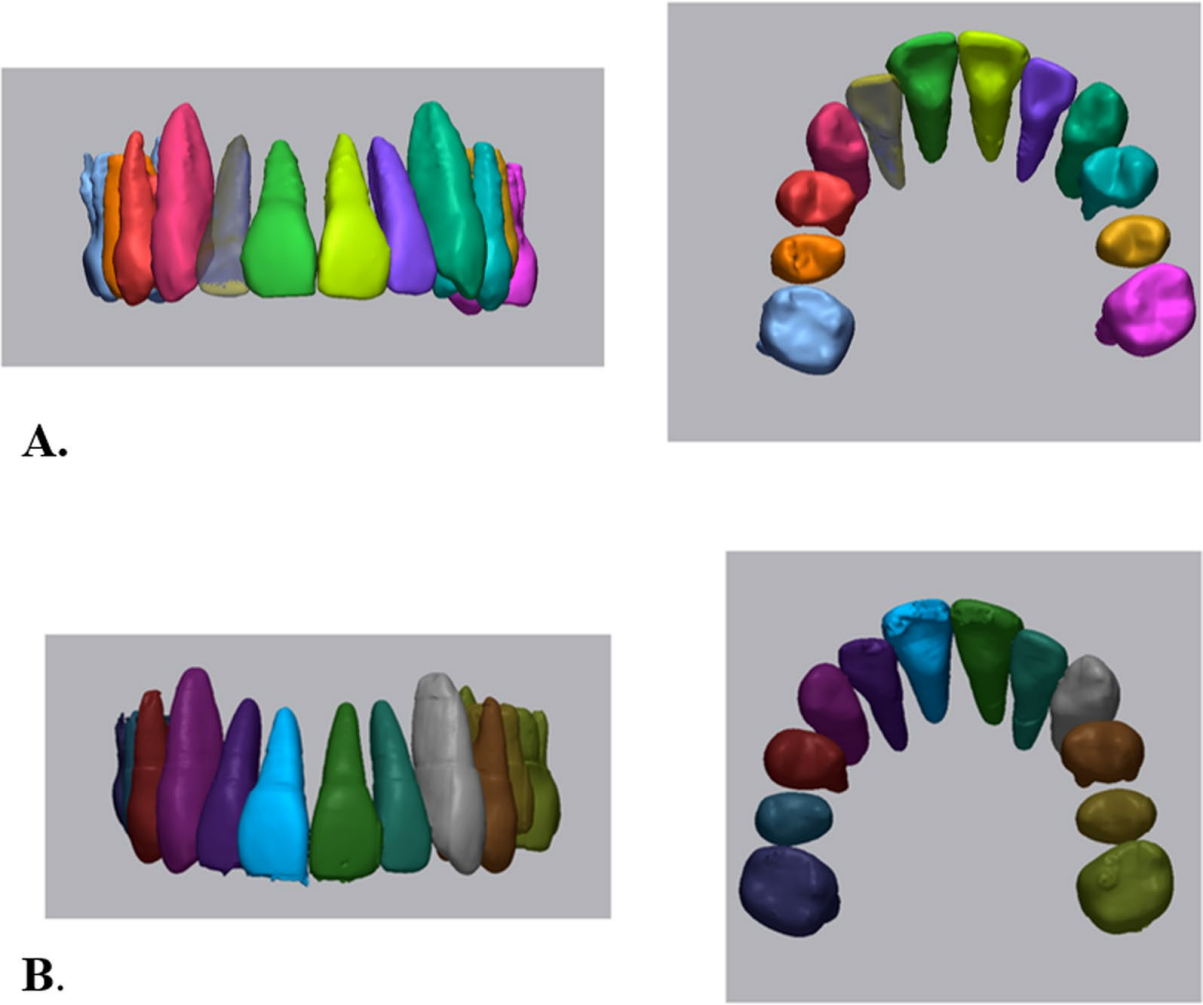
Tooth segmentation using manual (**a**) and deep learning (**b**) segmentation methods

To generate the 3D tooth model at pretreatment, intraoral scans and its corresponding CBCT scans at pretreatment are required. Intraoral scans of maxillary and mandibular arches were obtained using Trios scanner (3Shape, Copenhagen, Denmark). The intraoral scans were trimmed to the clinical crown by deleting the gingival area, and they were submitted to the OrthoAnalyzer (3Shape) program and reprocessed as a stereolithography (STL) file format. CBCT scans were taken with an Alphard Vega scanner (Asahi Roentgen Co. Kyoto, Japan) set at a field of view of 200 × 179 mm^2^, 80 kV, 5 mA, and a voxel size of 0.39 mm. Automatic and manual methods were used for the individual tooth segmentation for all teeth from the pretreatment and posttreatment CBCT scans. Mimics (version 23.0; Materialise, Leuven, Belgium) was used for manual method. Individual tooth segmentation was performed by extracting and isolating individual teeth from the surrounding alveolar bone using *region-growing* tool in the program. For automatic method, 3D tooth modeling using convolutional neural network (CephX, ORCA Dental AI Inc., Herzliya, Israel) was used to generate all teeth segmentations from pretreatment and posttreatment CBCT scans (Fig. 2).

Intraoral scans and segmented individual CBCT teeth were both imported into the 3D reverse engineering software (Rapidform 2006, INUS Technology, Seoul, Korea), and the 3D tooth model at pretreatment was fabricated through the integration process of the pretreatment CBCT-scanned tooth root and intraoral-scanned tooth crown. The first step in this integration process was to use “initial registration” function to grossly pick three corresponding points on both the crown of the pretreatment CBCT tooth and its corresponding pretreatment intraoral scans. The central fossa of the right and left second molars and midpoint of central incisors were selected as the three corresponding points. To maintain the orientation of CBCT scans, pretreatment CBCT teeth were locked in the software. Then, a “regional registration” function was used to finalize the best-fit registration, which uses an iterative closest algorithm. The regions of interest include labial surfaces of six anteriors and buccal surface of molars. Next, by changing the color opacity and being translucent, the crown area of CBCT tooth was erased to be replaced with its corresponding intraoral scan. Finally, the 3D tooth model, composed of CBCT-scanned root and intraoral-scanned crown, was generated by integrating these two imaging modalities using the “merge” function.

To estimate the posttreatment root position, individual tooth model at pretreatment was superimposed to the posttreatment intraoral scans. Both the individual tooth models at pretreatment and posttreatment intraoral scans were imported into the software. Initial registration and regional registration processes were used to superimpose two imaging modalities. Since the crown morphology is identical in principle, the only initial registration using picking several points was enough, but regional registration was added for a more accurate superimposition process.

**Fig. 3:**
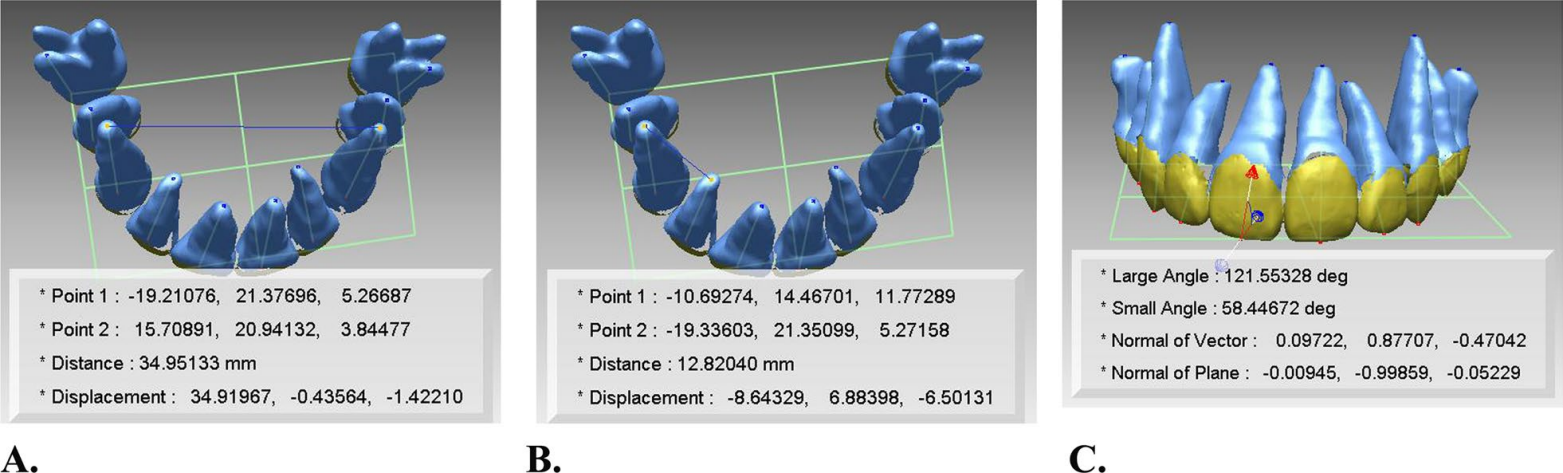
Measurements used for evaluation of integration accuracy. **a** and **b** 3D Euclidean arch width and inter-root distances in each estimated and actual tooth models; **c** root axis angle to occlusal plane in each estimated and actual tooth models

To determine whether the resulting estimated position of the root would agree with the actual root position at posttreatment CBCT, the discrepancy of the estimated and actual root position was calculated. First, the average surface discrepancy of estimated and actual tooth model in each automatic and manual method was calculated. Furthermore, the average surface discrepancy between the estimated and actual tooth models was compared between automatic and manual methods. For quantitative evaluation, 3D Euclidean inter-root distances between proximal teeth, arch widths, and root axis angle to occlusal plane were obtained from the estimated and actual tooth models, respectively. 3D Euclidean inter-root distances were obtained from distances between proximal teeth and arch widths were obtained from inter-incisor width, inter-canine width, inter-premolar width, and inter-molar width (Fig. 3). The distances were obtained by using the function of point-to-point distances in the software, and root apices were used for these points. Moreover, these values were compared between automatic and manual methods.

Furthermore, the difference between automatic and manual methods in terms of individual tooth segmentation was evaluated. The time taken to obtain these measurements was also recorded and compared between both methods. All these processes were conducted by an experienced single researcher with over 10 years of experience in this field, who graduated from a dental school and completed a Ph.D. program in orthodontics.

### Statistical analysis

The means and standard deviations of the measurements in the estimated and actual tooth models and manual and automatic methods. Shapiro–Wilk test for normal distribution of the differences accepted the normality; thus, a paired *t* test was used to analyze the differences between the estimated and actual tooth models and between the manual and automatic methods. Statistical analysis was performed by using SPSS (version 26.0; IBM SPSS, Armonk, NY). Statistical significance was set at *p* < 0.05. Intra-examiner repeatability was evaluated using intraclass correlation (ICC) analysis by repeating all measurements from five randomly selected individuals after 4 weeks. The ICC values were 0.857–0.939 and 0.820–0.913 in the maxillary and mandibular arches, respectively.

## Results

The average surface differences between estimated and actual ITMs in the manual method were 0.02 mm and 0.03 mm for the maxillary and mandibular arches, respectively. In the automatic method, the discrepancies were 0.07 mm and 0.08 mm for the maxillary and mandibular arches, respectively. However, there was no significant difference between the two methods (Table 1).

**Table 1:** Average surface difference of estimated and actual tooth model in each deep learning and manual method and its comparison between the two methods (unit: mm)

Average surface difference	Deep learning method	Manual method	*P* value*
	Mean ± SD	Mean ± SD	
Maxilla	0.07 ± 0.06	0.02 ± 0.02	0.863
Mandible	0.08 ± 0.04	0.03 ± 0.02	0.775

Data show the average surface discrepancy (mm) between the estimated and actual model in each deep learning and manual method by means of shell/shell deviation in the program. SD, Standard deviation.

*The results of paired *t* test

For the measurements of arch width, inter-root distance, and root axis angle, there were no significant differences between the estimated and actual models both in the manual and in the automatic methods except for some measurements (Tables 2, 3, 4, 5, 6, and 7). The premolar widths in both maxilla and mandible showed significant differences between estimated and actual models by means of the manual method. The difference of inter-premolar width between the estimated and actual models was 1.1 mm and 1.7 mm in the maxilla and mandible, respectively (Table 2). The inter-root distance between mandibular second premolar and canine showed a significant difference of 1.0 mm between the estimated and actual models by means of automatic method (Table 5). The root axis angle of maxillary first molar and mandibular incisors showed significant differences between the estimated and actual models by means of the automatic method. The difference was 2.4° in the maxillary first molar and 2.8° in the mandibular incisors (Table 7).

**Table 2:** Comparison of the arch width between estimated and actual tooth models in manual method (unit: mm)

	Estimated tooth model				Actual tooth model				*P *value*
	Mean	SD	Max	Min	Mean	SD	Max	Min	
Maxilla									
Inter-incisor width U1	6.8	1.3	8.8	5.2	7.1	1.0	8.4	6.1	0.384
Inter-incisor width U2	16.6	2.0	19.9	14.9	17.4	2.3	20.6	14.6	0.170
Inter-canine width	32.3	1.9	35.0	30.0	32.0	1.9	35.0	30.0	0.233
Inter-premolar width	38.6	2.8	40.4	33.8	39.7	2.7	42.1	35.4	0.014*
Inter-molar width	50.3	2.8	52.2	45.4	51.2	3.0	54.5	46.4	0.145
Mandible									
Inter-incisor width L1	4.0	1.6	6.7	2.8	4.0	1.2	5.9	2.9	0.946
Inter-incisor width L2	11.2	0.9	12.2	9.9	11.6	1.0	12.5	10.5	0.203
Inter-canine width	21.9	2.4	25.8	19.8	22.0	2.2	25.4	19.7	0.934
Inter-premolar width	30.3	1.6	33.0	29.0	32.0	2.2	35.1	29.7	0.018*
Inter-molar width	39.9	2.0	43.3	38.3	41.0	2.2	43.8	38.3	0.202

SD, Standard deviation

*The results of paired *t* test. U1, maxillary central incisors; U2, maxillary lateral incisors; L1, mandibular central incisors; L2, mandibular lateral incisors

**Table 3:** Comparison of the arch width between estimated and actual tooth models in deep learning method (unit: mm)

	Estimated tooth model				Actual tooth model				*P *value*
	Mean	SD	Max	Min	Mean	SD	Max	Min	
Maxilla									
Inter-incisor width U1	7.2	1.0	8.7	6.3	7.1	0.9	8.4	6.2	0.882
Inter-incisor width U2	17.6	1.8	20.1	15.7	18.1	2.2	20.3	15.3	0.488
Inter-canine width	32.5	2.3	36.0	30.1	32.1	2.5	35.8	29.3	0.366
Inter-premolar width	39.0	2.9	41.9	34.6	39.0	3.3	42.3	34.7	0.904
Inter-molar width	50.4	2.6	52.1	46.1	50.2	3.1	52.9	45.5	0.663
Mandible									
Inter-incisor width L1	4.2	1.3	6.2	2.9	4.3	1.0	5.8	3.3	0.883
Inter-incisor width L2	12.3	0.6	13.0	11.5	12.6	0.8	13.6	11.6	0.324
Inter-canine width	22.4	2.6	26.4	19.5	23.2	2.1	26.5	21.2	0.199
Inter-premolar width	31.1	1.7	33.6	29.6	31.9	1.8	34.8	30.1	0.115
Inter-molar width	38.9	2.5	42.5	36.3	39.9	2.0	41.6	37.3	0.316

SD, Standard deviation

*The results of paired *t* test. U1, maxillary central incisors; U2, maxillary lateral incisors; L1, mandibular central incisors; L2, mandibular lateral incisors

Comparing the manual and automatic methods, only three measurements, namely inter-root distance between right maxillary first molar and second premolar, left maxillary canine and lateral incisor, and root axis angle of mandibular incisors, showed significant difference between the two methods (Tables 8, 9, and 10).

**Table 4:** Comparison of inter-root distance between estimated and actual tooth models in manual method (unit: mm)

	Estimated tooth model				Actual tooth model				*P *value*
	Mean	SD	Max	Min	Mean	SD	Max	Min	
*Maxilla, Rt*									
Inter-root distances between U6 and U5	9.4	1.0	10.6	8.0	8.8	0.7	9.6	8.1	0.138
Inter-root distances between U5 and U3	6.4	0.9	7.3	5.1	6.8	0.7	7.6	6.0	0.089
Inter-root distances between U3 and U2	10.7	2.0	12.6	8.6	10.5	0.8	11.7	9.8	0.709
Inter-root distances between U2 and U1	6.6	2.4	9.7	4.3	6.3	1.4	8.1	5.0	0.535
Maxilla, *Lt*									
Inter-root distances between U6 and U5	8.6	0.8	9.9	7.7	8.8	0.5	9.3	8.3	0.531
Inter-root distances between U5 and U3	4.9	1.8	7.6	2.5	5.3	1.4	6.8	3.7	0.421
Inter-root distances between U3 and U2	9.6	2.5	12.9	6.1	9.8	2.0	11.8	6.5	0.673
Inter-root distances between U2 and U1	6.4	2.2	9.8	4.1	5.9	1.4	7.7	4.1	0.408
Mandible, *Rt*									
Inter-root distances between L6 and L5	7.4	1.3	8.6	5.2	7.3	0.9	8.5	6.4	0.834
Inter-root distances between L5 and L3	5.9	0.7	6.7	5.2	6.7	1.4	9.0	5.5	0.165
Inter-root distances between L3 and L2	6.7	1.1	8.2	5.4	6.8	1.0	7.8	5.6	0.537
Inter-root distances between L2 and L1	4.7	1.5	6.9	3.2	4.3	0.7	5.3	3.7	0.338
Mandible, *Lt*									
Inter-root distances between L6 and L5	8.2	1.5	9.8	5.9	7.7	1.5	9.7	5.5	0.072
Inter-root distances between L5 and L3	6.5	1.3	8.5	5.5	7.2	1.3	9.1	6.0	0.141
Inter-root distances between L3 and L2	7.2	1.2	9.3	6.2	7.1	1.4	8.9	5.5	0.702
Inter-root distances between L2 and L1	4.5	0.4	5.1	4.1	4.3	0.5	4.8	3.5	0.410

SD, Standard deviation

*The results of paired *t* test. U6, maxillary first molar; U5, maxillary second premolar; U3, maxillary canine; U2, maxillary lateral incisor; U1, maxillary central incisor. L6, mandibular first molar; L5, mandibular second premolar; L3, mandibular canine; L2, mandibular lateral incisor; L1, mandibular central incisor

**Table 5:** Comparison of inter-root distance between estimated and actual tooth models in deep learning method (unit: mm)

	Estimated tooth model				Actual tooth model				*P *value*
	Mean	SD	Max	Min	Mean	SD	Max	Min	
Maxilla, *Rt*									
Inter-root distances between U6 and U5	8.6	1.1	9.8	7.0	8.6	0.7	9.4	7.8	0.935
Inter-root distances between U5 and U3	6.4	1.0	7.6	5.2	6.5	1.0	7.9	5.4	0.476
Inter-root distances between U3 and U2	10.1	1.4	12.0	8.6	10.1	0.8	11.4	9.5	0.930
Inter-root distances between U2 and U1	6.8	1.8	9.1	5.0	6.8	1.3	8.3	5.6	0.869
Maxilla, *Lt*									
Inter-root distances between U6 and U5	8.1	0.5	8.7	7.5	9.1	0.7	10.1	8.0	0.065
Inter-root distances between U5 and U3	5.7	1.4	7.6	4.2	5.6	1.4	6.9	3.4	0.618
Inter-root distances between U3 and U2	9.6	2.2	12.5	6.5	9.4	1.8	11.1	6.7	0.670
Inter-root distances between U2 and U1	6.3	1.4	8.1	4.5	6.2	1.2	7.7	4.9	0.780
Mandible, *Rt*									
Inter-root distances between L6 and L5	7.0	1.2	8.3	5.7	7.0	0.7	7.9	6.1	0.882
Inter-root distances between L5 and L3	6.2	0.5	6.7	5.4	6.7	1.3	8.9	5.6	0.353
Inter-root distances between L3 and L2	7.2	1.1	8.6	6.0	6.8	0.8	8.1	6.1	0.159
Inter-root distances between L2 and L1	5.1	1.1	6.3	3.4	4.4	0.4	5.0	3.9	0.227
Mandible, *Lt*									
Inter-root distances between L6 and L5	7.8	1.3	9.1	5.7	7.4	1.4	9.3	5.3	0.128
Inter-root distances between L5 and L3	6.0	1.2	7.6	4.8	7.0	1.7	9.2	5.3	0.042*
Inter-root distances between L3 and L2	7.9	1.0	9.1	6.9	7.1	1.1	8.7	5.8	0.149
Inter-root distances between L2 and L1	4.6	0.3	4.9	4.1	4.8	0.5	5.3	4.0	0.714

SD, Standard deviation

*The results of paired *t* test. U6, maxillary first molar; U5, maxillary second premolar; U3, maxillary canine; U2, maxillary lateral incisor; U1, maxillary central incisor. L6, mandibular first molar; L5, mandibular second premolar; L3, mandibular canine; L2, mandibular lateral incisor; L1, mandibular central incisor

**Table 6:** Comparison of root axis angle to occlusal plane between estimated and actual tooth models in manual method (unit: degree)

	Estimated tooth model				Actual tooth model				P value*
	Mean	SD	Max	Min	Mean	SD	Max	Min	
Maxilla									
Incisors	58.4	4.4	70.3	49.0	58.8	3.9	67.2	52.7	0.461
Canine	67.0	4.1	72.0	58.4	67.3	3.7	73.4	62.7	0.763
Second premolar	78.6	4.5	86.3	69.6	78.5	5.4	86.0	71.8	0.949
First molar	79.4	4.4	87.1	74.4	79.8	5.4	88.5	73.7	0.741
Mandible									
Incisors	58.4	5.1	70.5	51.7	58.6	5.0	68.8	52.2	0.648
Canine	67.5	5.4	77.0	62.0	66.9	6.0	76.9	60.3	0.294
Second premolar	74.8	4.1	82.4	67.8	74.0	3.1	78.5	68.3	0.468
First molar	76.8	5.1	86.2	68.4	77.7	4.9	86.5	68.7	0.338

SD, Standard deviation

*The results of paired *t* test

**Table 7:** Comparison of root axis angle to occlusal plane between estimated and actual tooth models in deep learning method (unit: degree)

	Estimated tooth model				Actual tooth model				*P *value*
	Mean	SD	Max	Min	Mean	SD	Max	Min	
Maxilla									
Incisors	59.0	3.9	68.2	51.6	59.5	2.9	67.1	54.7	0.455
Canine	68.5	4.1	74.2	60.4	68.1	3.4	72.3	61.7	0.522
Second premolar	78.4	5.8	87.4	70.7	77.7	5.1	85.0	71.1	0.425
First molar	82.6	3.4	88.2	79.2	80.2	3.1	83.4	75.1	0.021*
Mandible									
Incisors	60.1	6.0	72.0	50.4	62.9	4.5	71.5	54.4	0.001*
Canine	69.4	5.7	80.3	63.9	70.5	4.7	76.8	65.0	0.217
Second premolar	77.9	4.0	84.8	70.9	76.2	3.7	80.0	69.3	0.213
First molar	79.5	4.7	86.6	72.4	81.0	3.5	86.5	76.7	0.370

SD, Standard deviation

*The results of paired *t* test

**Table 8:** Comparison of the difference of estimated and actual tooth models in arch width between manual and deep learning methods (unit: mm)

Difference of estimated and actual tooth models	Manual method	Deep learning method	*P *value*
	Mean ± SD	Mean ± SD	
Maxilla			
Inter-incisor width U1	− 0.32 ± 0.73	− 0.06 ± 0.84	0.238
Inter-incisor width U2	− 0.84 ± 1.13	− 0.84 ± 1.13	0.184
Inter-canine width	0.34 ± 0.54	0.36 ± 0.73	0.966
Inter-premolar width	− 1.12 ± 0.60	− 0.06 ± 1.05	0.079
Inter-molar width	− 0.90 ± 1.11	0.20 ± 0.95	0.077
Mandible			
Inter-incisor width L1	0.02 ± 0.62	0.02 ± 0.29	0.088
Inter-incisor width L2	− 0.44 ± 0.64	− 0.32 ± 0.63	0.695
Inter-canine width	− 0.04 ± 1.02	− 0.76 ± 1.10	0.208
Inter-premolar width	− 1.72 ± 0.90	− 0.80 ± 0.89	0.172
Inter-molar width	− 1.10 ± 1.61	− 1.04 ± 2.03	0.920

SD, Standard deviation

*The results of paired *t* test. U1, maxillary central incisors; U2, maxillary lateral incisors; L1, mandibular central incisors; L2, mandibular lateral incisors

**Table 9:** Comparison of the difference of estimated and actual tooth models in inter-root distance between manual and deep learning methods (unit: mm)

Difference of estimated and actual tooth models	Manual method	Deep learning method	*P *value*
	Mean ± SD	Mean ± SD	
Maxilla, *Rt*			
Inter-root distances between U6 and U5	0.54 ± 0.65	− 0.02 ± 0.52	0.008*
Inter-root distances between U5 and U3	− 0.40 ± 0.40	− 0.12 ± 0.34	0.058
Inter-root distances between U3 and U2	0.26 ± 1.45	0.04 ± 0.96	0.453
Inter-root distances between U2 and U1	0.34 ± 1.12	− 0.06 ± 0.76	0.222
Maxilla, *Lt*			
Inter-root distances between U6 and U5	− 0.22 ± 0.72	− 0.94 ± 0.83	0.053
Inter-root distances between U5 and U3	− 0.46 ± 1.15	0.14 ± 0.58	0.082
Inter-root distances between U3 and U2	− 0.18 ± 0.88	0.18 ± 0.87	0.011*
Inter-root distances between U2 and U1	0.46 ± 1.11	0.10 ± 0.75	0.276
Mandible, *Rt*			
Inter-root distances between L6 and L5	0.08 ± 0.80	− 0.04 ± 0.56	0.761
Inter-root distances between L5 and L3	− 0.78 ± 1.02	− 0.56 ± 1.19	0.360
Inter-root distances between L3 and L2	− 0.10 ± 0.33	0.42 ± 0.54	0.146
Inter-root distances between L2 and L1	0.42 ± 0.86	0.66 ± 1.03	0.614
Mandible, *Lt*			
Inter-root distances between L6 and L5	0.54 ± 0.49	0.34 ± 0.39	0.368
Inter-root distances between L5 and L3	− 0.78 ± 0.95	− 1.06 ± 0.80	0.636
Inter-root distances between L3 and L2	0.16 ± 0.87	0.74 ± 0.92	0.278
Inter-root distances between L2 and L1	0.22 ± 0.53	− 0.12 ± 0.68	0.401

SD, Standard deviation

*The results of paired *t* test. U6, maxillary first molar; U5, maxillary second premolar; U3, maxillary canine; U2, maxillary lateral incisor; U1, maxillary central incisor. L6, mandibular first molar; L5, mandibular second premolar; L3, mandibular canine; L2, mandibular lateral incisor; L1, mandibular central incisor

**Table 10:** Comparison of the difference of estimated and actual tooth models in root axis angle to occlusal plane between manual and deep learning methods (unit: degree)

Difference of estimated and actual tooth models	Manual method	Deep learning method	*P *value*
	Mean ± SD	Mean ± SD	
Maxilla			
Incisors	− 0.44 ± 2.61	− 0.43 ± 2.55	0.992
Canine	− 0.27 ± 2.75	0.39 ± 1.85	0.533
Second premolar	0.06 ± 2.89	0.68 ± 2.57	0.554
First molar	− 0.45 ± 4.18	2.41 ± 2.74	0.088
Mandible			
Incisors	− 0.19 ± 1.83	− 2.81 ± 2.76	0.002*
Canine	0.64 ± 1.81	− 1.09 ± 2.59	0.090
Second premolar	0.82 ± 3.42	1.70 ± 4.01	0.200
First molar	− 0.92 ± 2.87	− 1.50 ± 5.02	0.714

SD, Standard deviation

*The results of paired *t* test

The procedure time taken to obtain the measurements was longer in the manual method than in the automatic method. Individual tooth segmentation using Mimics program took 15 min for each tooth, and 24 teeth including first molar required 6 h. Individual tooth segmentation using automatic method took 1 min for each tooth, and 24 teeth including first molar required 24 min without manual labor.

## Discussion

Lee et al. [13, 14, 16, 17] attempted to combine a CBCT image and laser-scanned models for evaluating the root position at different stages of orthodontic treatment and reported that tooth root position could be predicted from combination with pretreatment CBCT images and posttreatment laser-scanned model images. However, pretreatment CBCT images do not provide detailed information of crown morphology and occlusion. When interdigitation between maxillary and mandibular dentition is tight or crown restorations are present, artifacts of CBCT images occur; thus, it is difficult to integrate the two imaging modalities, i.e., CBCT and laser-scanned model imaging. Inaccuracy of integration of two imaging modalities affects final predictability of tooth root position. Therefore, in this report, from the beginning stage before orthodontic treatment, pretreatment 3D tooth models were generated from pretreatment CBCT and pretreatment intraoral scans. The tooth roots were obtained from the CBCT image, and the tooth crowns were obtained from the intraoral scans, thereby minimizing the possibility of overlapping error (integration error) due to the artifacts of the crown appearing in the CBCT image at the pretreatment stage.

Tooth segmentation is an important step for fabricating the individual tooth model, of which accurate segmentation is essential. Various computer algorithms for automatic tooth segmentation have been proposed, and some software programs for automatic segmentation have been released in dentistry. Hence, a method that isolates the tooth including the root from the alveolar bone in CBCT images without removing the alveolar bone is preferable. The software used in this study is generally used for processing medical images and creating 3D models. Unlike medical segmentation process of other anatomic structures such as the pelvic bone or heart, tooth segmentation from alveolar bone was difficult. Basically, the program performs segmentation by differentiating and taking different levels of multiple anatomic structures. However, the contrast level of the tooth and alveolar bones is similar, and differentiating between the two is hard for the software due to very narrow periodontal ligament space between tooth and alveolar bone. Thus, fully automatic segmentation was impossible for isolating tooth from the alveolar bone. The program's automatic segmentation function, *region-growing*, was primarily used for rough segmentation, and it was adjusted manually for accuracy using the *slice edit* tool. The *region-growing* tool provides the capacity to split the segmentation into separate objects. *Morphology operation* prior to *region-growing* was done on all slices to take the intrinsic tooth structure from the bone. There are two options in the function for 8-connectivity and 26-connectivity; 26-connectivity was applied to select the pixels in the boundary of the structure considering neighboring pixels in 3D. One study about the segmentation method of watershed transformation reported that the proper selection of the segmentation threshold is critical for CBCT images with a low contrast and high noise level [18]. Ye et al. [9] evaluated the integration accuracy of CBCT images and dental model according to segmentation threshold settings, and they found that the accuracy of the integration of laser-scanned dental models into CBCT images is higher with a high-relative Hounsfield unit threshold setting in 0.20 and 0.40 mm voxel sizes [9].

In order to estimate the posttreatment root position, individual tooth model at pretreatment was superimposed to the posttreatment intraoral scans. In other words, pretreatment individual tooth model which was fabricated by combining pretreatment intraoral scan and CBCT data was replaced with a posttreatment intraoral scan. Since the crown morphology is identical in pretreatment and posttreatment intraoral scan, the root position would be changed according to the position of posttreatment intraoral scan. Then, this changed root position was compared with the actual root position at posttreatment CBCT. The actual tooth model means the tooth model was fabricated using posttreatment CBCT data in each method. In other words, these actual tooth models were fabricated in each method; thus, there was a slight difference between the actual tooth models from each method.

In Table 2, the values of estimated models were smaller than those of actual tooth models. All but two of the estimated inter-arch widths were underestimated via the estimate model using the manual method. In contrast, in Table 3, using the deep learning method, three of the five maxillary inter-arch measures were over-estimated. The mandibular inter-arch measures were similar to the manual method where the estimated tooth model also underestimates the actual model. The possible reason of that was morphological changes of root apex after treatment. The actual models were fabricated using CBCT scan after treatment. The reason why there were underestimated values ​​in the maxilla was that the morphological changes of root apex occurred more in the maxilla than in the mandible.

In the present study, there were differences around the second premolar area between estimated and actual tooth models. All study participants with premolar extraction had undergone first premolar extraction. The amount of tooth movement is generally large around extraction spaces. It is believed that these differences are due to the large amount of tooth movement in the second premolar area. Moreover, the root axis angle of mandibular incisors showed significant differences between the estimated and actual models using the automatic method. The root axis angle of mandibular incisors also showed significant differences in the comparison between the manual and automatic methods. The inter-radicular spaces around the mandibular incisors are narrow; thus, inter-radicular alveolar bone is commonly thin around the mandibular incisors. This might lead to errors in tooth segmentation process. Therefore, careful consideration of tooth segmentation is essential for this area.

In this study, the second molars were excluded, as they are rarely monitored compared to other teeth during orthodontic treatment. In addition, there are many cases in which the tooth root morphology of the second molars rather than the first molars is irregular, increasing the likelihood of errors. Furthermore, intraoral scanning inaccuracy of second molars may also lead to errors [19, 20]. However, as digital technologies in orthodontic treatment including virtual setup and indirect bonding using 3D printing have become popular, the second molars are included in orthodontic treatment from the beginning of the treatment stages. Considering this, further research including the measurements of second molars is necessary.

Regarding the reproducibility of the fabrication process of tooth models, accuracy of tooth models may be affected by the construction skill and experience of the examiners. In the present study, all processes were conducted by an experienced single researcher with over 10 years of experience in this field, who graduated from a dental school and completed a Ph.D. program in orthodontics for minimizing reproducibility errors. The tooth-modeling service of CephX was used for the deep learning automatic method using convolutional neural network features. With the advent of deep learning technology, artificial intelligence technology is showing remarkable practical effects, as it can analyze and learn like a human; recognize data in text, image, and sound format; and perform image classification, segmentation, and enhancement.

In the present study, the 3D reverse engineering software (Rapidform) was used for the integration process of the pretreatment CBCT-scanned tooth root and intraoral-scanned tooth crown. For other currently available software, there are Dental Monitoring and Geomagic software (3D Systems, USA). Geomagic software mainly provides the ability to process STL or computer-aided design (CAD) file format and is commonly used in the fields of making digital 3D models and CAD assemblies. One study regarding the accuracy of Dental Monitoring application reported that 3D digital dental models generated by the Dental Monitoring application in photograph and video modes were accurate enough to be used for clinical applications [21].

Deep learning offers advantages in reading medical images and diagnosing diseases. Artificial intelligence technology brings forth novel diagnostic and therapeutic systems for radiology, imaging technology, ultrasonography, and pathological diagnosis, which can improve the quality and efficiency of clinical work comprehensively. Moreover, the technology is gradually changing the traditional medical model, representing a direction and trend for future human medical development.

## Conclusion

Both the automatic and manual methods showed similar accuracy in the integration of intraoral scans and CBCT images. Considering time and efficiency, the automatic method for ITMs is highly recommended for clinical practice.

## Data Availability

The data and materials obtained in this study belong to the authors and are therefore available only upon request, after approval by the authors.

## Abbreviations

ITM: Integrated tooth model
3D: Three-dimensional
CBCT: Cone-beam computed tomography
STL: Stereolithography
CAD: Computer-aided design
